# SLIM: a flexible web application for the reproducible processing of environmental DNA metabarcoding data

**DOI:** 10.1186/s12859-019-2663-2

**Published:** 2019-02-19

**Authors:** Yoann Dufresne, Franck Lejzerowicz, Laure Apotheloz Perret-Gentil, Jan Pawlowski, Tristan Cordier

**Affiliations:** 1 Department of Genetics and Evolution, University of Geneva, Science III, 4 Boulevard d’Yvoy, 1205 Geneva, Switzerland; 2 Institut Pasteur, Bioinformatics and Biostatistics Hub, C3BI, Paris, France; 3 Department of Computer Science and Engineering, University of California San Diego, San Diego, California USA

**Keywords:** eDNA metabarcoding, High-throughput sequencing, Molecular ecology, Pipeline, Reproducibility, Amplicon sequencing

## Abstract

**Background:**

High-throughput amplicon sequencing of environmental DNA (eDNA metabarcoding) has become a routine tool for biodiversity survey and ecological studies. By including sample-specific tags in the primers prior PCR amplification, it is possible to multiplex hundreds of samples in a single sequencing run. The analysis of millions of sequences spread into hundreds to thousands of samples prompts for efficient, automated yet flexible analysis pipelines. Various algorithms and software have been developed to perform one or multiple processing steps, such as paired-end reads assembly, chimera filtering, Operational Taxonomic Unit (OTU) clustering and taxonomic assignment. Some of these software are now well established and widely used by scientists as part of their workflow. Wrappers that are capable to process metabarcoding data from raw sequencing data to annotated OTU-to-sample matrix were also developed to facilitate the analysis for non-specialist users. Yet, most of them require basic bioinformatic or command-line knowledge, which can limit the accessibility to such integrative toolkits. Furthermore, for flexibility reasons, these tools have adopted a step-by-step approach, which can prevent an easy automation of the workflow, and hence hamper the analysis reproducibility.

**Results:**

We introduce SLIM, an open-source web application that simplifies the creation and execution of metabarcoding data processing pipelines through an intuitive Graphic User Interface (GUI). The GUI interact with well-established software and their associated parameters, so that the processing steps are performed seamlessly from the raw sequencing data to an annotated OTU-to-sample matrix. Thanks to a module-centered organization, SLIM can be used for a wide range of metabarcoding cases, and can also be extended by developers for custom needs or for the integration of new software. The pipeline configuration (i.e. the modules chaining and all their parameters) is stored in a file that can be used for reproducing the same analysis.

**Conclusion:**

This web application has been designed to be user-friendly for non-specialists yet flexible with advanced settings and extensibility for advanced users and bioinformaticians. The source code along with full documentation is available on the GitHub repository (https://github.com/yoann-dufresne/SLIM) and a demonstration server is accessible through the application website (https://trtcrd.github.io/SLIM/).

## Background

High-throughput amplicon sequencing of environmental DNA (eDNA metabarcoding) is a fast and affordable molecular approach to monitor biodiversity [1]. Metabarcoding has indeed become a routinely used tool for various ecological field, such as terrestrial and marine biodiversity studies [2], animals diet survey [3] or biomonitoring [4–6]. It has even proved useful for paleo-environmental events detection [7], archeological studies [8], and the detection of airborne pollen [9]. The data generated by sequencing platforms during these studies is being processed by a succession of software (a so-called pipeline) to translate the raw sequences (or reads) into a statistically exploitable contingent matrix that contains Operational Taxonomic Units (OTU) as rows and samples as columns (i.e. the so-called “OTU-table”). These processing steps are indeed critical for accurate biological interpretation [10–13].

The metabarcoding processing steps can be broadly grouped in five categories:

1. Demultiplexing samples: Most of the metabarcoding studies uses multiplexing for a better cost-effectiveness, i.e. including sample-specific tags in the primers prior PCR amplification [14]. From a given multiplexed library (pooled PCR products with unique adaptors pairs at both 3′ and 5′ ends of the reads), multiple samples need to be retrieved and “demultiplexed” into separate sample-specific files. In the case of each library represents a unique sample, this step can be ignored.
2. Reads joining: For paired-end sequencing data, reads need to be joined into full-length contigs. This step can also be seen as quality filtering, because non-overlapping reads are being discarded. For single-end sequenced libraries, this step can be ignored.
3. Quality filtering: It regroups multiple type of filters, including base-calling quality filters, PCR and sequencing errors denoiser [15] or chimera filter [16]. This step is crucial to remove as much technical noise as possible in the data.
4. OTU clustering: This step has received most of the attention and is still an active field of bioinformatic research. The sequences are grouped by similarity into clusters that represent proxies for molecular species (de novo OTU clustering strategy). Open or closed reference OTU clustering strategies sequences are mapped represent alternatives (sequences are first clustered against a reference sequence database), even though they have been shown to be outperformed by de novo approaches in some cases [17]. This step is critical to yield a maximum of biologically relevant information and has a strong impact on diversity measures and downstream analysis.
5. Taxonomic assignment: Putatively ascribe a taxonomic name to each OTU. Curated sequence databases such as SILVA [18] or PR2 [19] for nuclear ribosomal markers, BOLD [20] or MIDORI [21] for cytochrome oxidase I or UNITE for fungal Internal Transcribed Spacer [22] can be used as reference. Important efforts are made to improve methods and algorithms for more accurate taxonomic assignment, and various approaches have been explored [23–26].

Multiple algorithms and software have been developed to perform one or multiple processing steps. They can be called sequentially via command-line or bash scripts to form an analysis pipeline, provided that the input/output file format between each of these software is handled correctly. Wrappers and toolkits such as MOTHUR [27], USEARCH [28], QIIME [29], OBITools [30] or VSEARCH [31] have been developed specifically for routine analysis of eDNA metabarcoding data. However, non-specialists or command-line reluctant users might still not feel comfortable. Moreover, users are often left to find by themselves a relevant traceability system for their analysis, which can hamper the analyses reproducibility. The software galaxy [32] was developed to allow users to create their own pipelines through a web Graphical User Interface (GUI). However, it has been designed to remain as broad as possible in term of application. This means going through a long configuration and installation step prior any data analysis. A command-line free tool specifically designed for metabarcoding studies, yet flexible and powerful, would allow every scientist working with such sequencing data to be autonomous for the carry-out of these critical processing steps.

Here, we introduce SLIM, an open-source web application for the reproducible processing of metabarcoding data, from the raw sequences to an annotated OTU table. The application is meant to be deployed on a local computing server or on personal computers for users without internet connection or developers. We provide a demonstration version of SLIM with reduced computing capacity, accessible through the application website (https://trtcrd.github.io/SLIM/).

## Implementation

### Overview

SLIM is an web-application with a Graphical User Interface (GUI) that help users to create and execute their own metabarcoding pipelines using state-of-art, open-source and well-established software. The core of SLIM is based on the Node JavaScript runtime, an open source server framework that have been designed for the building of scalable network applications, by handling asynchronous and parallel events. The installation is made as easy as possible for system administrators, through bash scripts that fetch the dependencies, and deploy the web application into a docker container (www.docker.com). This means that the application can be deployed on various platform, from a personal computer to a local or cloud-based computing server. The development of SLIM was guided by the four following principles:

#### Making it user-friendly for non-specialists

This involves creating a Graphical User Interface (GUI) to avoid the need of any command line. For Operating System (OS) cross compatibilities, portability and maintenance, we used web technologies (JavaScript, HTML and CSS) to build the GUI. Therefore, there is no need for any installation on user’s personal computers. Instead, SLIM is accessible through a web-browser over local network or over the internet, from any operating system (OS).

#### Making the installation and administration as easy as possible

To facilitate the installation and the deployment of SLIM by systems administrators while ensuring the security and stability of a computing server configuration, we embedded the application in a docker container (https://www.docker.com). Thanks to this solution, SLIM can be deployed on Unix-like OS (macOS and Linux). We created two bash scripts, one to fetch the application dependencies and another one to deploy it. The application is versioned and frozen into stable releases hosted in GitHub. Once deployed, SLIM includes a logging system that is accessible through docker commands.

#### Encouraging analysis reproducibility

Analysis reproducibility and transparency is a growingly recognized issue. We included an easy way to reproduce an analysis carried out by SLIM. Each execution, which includes a succession of software with their associated parameters can be saved and stored as a small configuration file. To exactly reproduce an execution, one just need the raw sequencing data, the stable version of SLIM that has been used and this configuration file.

#### Facilitating its extensibility

The integration of new software into modules has been made as easy as possible. It requires only some knowledge of web-based languages (JavaScript and HTML) and for input/output file format handling (usually done by python scripts). Once the set of module’s associated files are in place within the application folders, the integration itself is done automatically by the application core functions. Developers are encouraged to pull request their new modules and new features to the SLIM repository (https://github.com/yoann-dufresne/SLIM). These new features will be merged to SLIM and made available on the demonstration server.

### A module-centered application

**Fig. 1:**
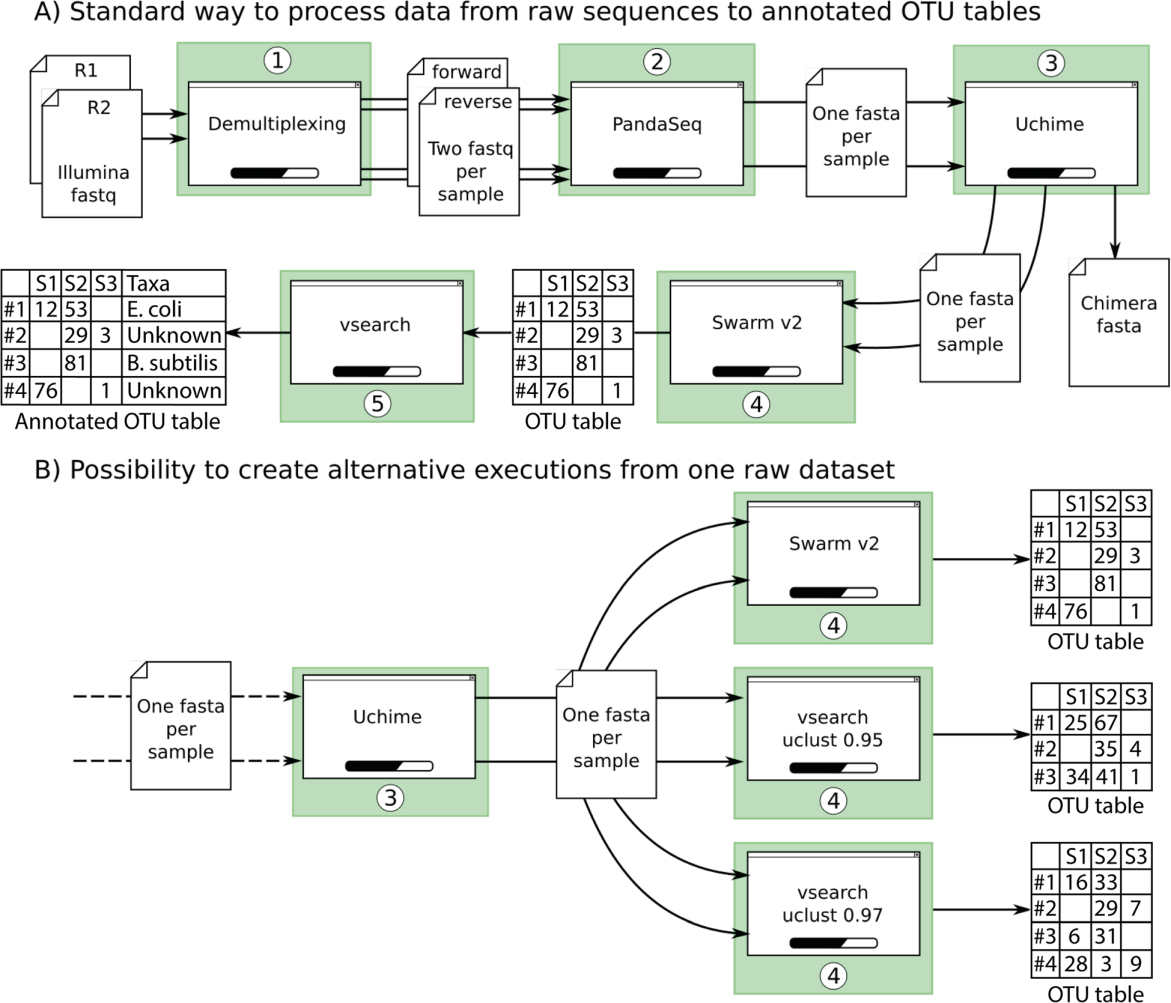
Two pipeline examples using SLIM. A) A commonly used workflow including usual processing steps, from the demultiplexing to an annotated OTU table. B) An alternate workflow using different OTU clustering strategies to assess the impact of this processing step on the biological conclusions

All the implemented software and tools are independently encapsulated in modules. Each module is defined with its input files, its parameters and its output files. This organization structure makes it possible to create single or parallel workflows to study the impact of a specific step on the biological conclusions, by connecting outputs of modules to inputs of others (Fig. 1). This chaining organization makes SLIM flexible and adapted for a wide range of use cases. Indeed, adding and chaining modules is an intuitive way to design workflows. The processing modules that are readily available in SLIM and the ones that are planned to be included in future development is listed in Table 1. These future modules include for instance a mistagging filter [33], the DADA2 [15] workflow for Amplicon Sequence Variant (ASV) inference, the CREST [24] and IDTAXA [34] taxonomic assignment method, the Short Read Archive (SRA) toolkit for fetching raw data directly from the application, but also some post-processing tools. For instance, the R package LULU that implement a post clustering curation algorithm [35] has been integrated, and the R package BBI for computing Biotic Indices from the taxonomic assignments [36] will be soon available. A complete documentation for each module specifications is available on the SLIM GitHub repository wiki. We also provide a detailed documentation for the development of new modules.

**Table 1:** List of available modules in SLIM and planned integration

Processing step	Module	Availability	References
SRA downloader	SRA	planned	Short Read Archive tools (https://github.com/ncbi/sra-tools)
Demultiplexing	DTD	yes	https://github.com/yoann-dufresne/DoubleTagDemultiplexer
Mistag-filtering	mistag	planned	[33]
Denoising / ASV inference	DADA2	planned	[15]
Reads joining	PANDAseq	yes	[37]
	CASPER	yes	[38]
	VSEARCH	yes	[31]
Chimera-removal	VSEARCH	yes	[31]
OTU clustering	VSEARCH	yes	[31]
	SWARM	yes	[39]
Taxonomic assignement	VSEARCH	yes	[31]
	CREST	planned	[24]
	IDTAXA	planned	[34]
Post-processing	LULU	yes	[35]
	Biotic Indices	planned	[36]

### The job execution, data management and queuing system

Once the data is uploaded and the pipeline has been set, users provide their email address and trigger the execution. An email containing a unique link to the job as well as the configuration file will be immediately sent to the user. The job will be automatically scheduled and run. As soon as the job is done, a second email will be sent, inviting the user to download the annotated OTU table and any intermediate file of interest. By default, the raw data and results file will remain available on the server for a period of 24 h after job completion for storage optimization.

The application has been designed to be multi-tenant and to adapt the number of parallel users (i.e. tenant) that can perform an execution to the computing capacity of the hosting machine. By default, we have set the application to execute a user’ job on up to 8 CPU cores (16 cores make it possible to execute two users’ job in parallel, etc.). If a new job is submitted while all the CPU cores are already busy, it will be queued. Queued jobs will be scheduled as soon as enough CPU cores become available.

## Results and discussion

SLIM is a user-friendly web application specifically designed for the processing of raw metabarcoding data to obtain annotated OTU tables. It simplifies the use of state-of-art bioinformatics tools, by providing an intuitive GUI that allows users to quickly design their own analysis pipeline. It also facilitates the reproducibility of a such analysis, by sending to the user an email containing a configuration file that includes all the pipeline settings. Hence, reproducing an analysis requires only the raw sequencing dataset, the version of SLIM that was used, and this configuration file. We think that including such configuration file as supplementary material in publications will contribute to improve the reproducibility of metabarcoding analysis.

Thanks to the use of web technologies, SLIM is cross-platform and is meant to be deployed on computing server and accessed remotely over local network or over the internet. However, users with limited internet connection and developers can also install the application on their own personal computer running Unix-like OS (Linux or macOS).

The future development and integration of new modules has been made as easy as possible and will make SLIM even more flexible and useful to the metabarcoding users community. This aspect is of prime importance as sequencing technologies are constantly being improved and keep in challenging our computing tools to extract biologically relevant information from this ever-growing amount of data.

## Conclusion

For demonstration purpose, a server is accessible from the project website hosted on GitHub (https://trtcrd.github.io/SLIM/) and has been restricted to process up to one single full illumina MiSeq platform run (approximately 15 million reads) or to execute quickly an analysis on a provided example dataset.

### Availability and requirements

Project name: SLIM

Project home page: https://github.com/yoann-dufresne/SLIM

Project demonstration page: https://trtcrd.github.io/SLIM/

Operating system(s): Linux, macOS

Programming language: JavaScript, Python, HTML, CSS, Shell

Other requirements: docker

License: AGPL v3

## Abbreviations

CPU: Computing processing unit
GUI: Graphical user interface
OTU: Operational taxonomic unit
PCR: Polymerase chain reaction
